# Cellular and nuclear morphology…and calcium signaling: revealing the interplay between structure and function

**DOI:** 10.1186/1471-2202-13-S1-P65

**Published:** 2012-07-16

**Authors:** Markus Breit, Peter Bengtson, Anna Hagenston, Hilmar Bading, Gillian Queisser

**Affiliations:** 1 Goethe Center for Scientific Computing, Computational Neuroscience Group, University of Frankfurt, Frankfurt am Main, 60325, Germany; 2 Department of Neurobiology, Interdisciplinary Center for Neuroscience, University of Heidelberg, Heidelberg, 68120, Germany

## 

Calcium plays a pivotal role in relaying electrical signals of the cell to subcellular compartments, such as the nucleus. Since this one ion type is used by the cell for many processes a neuron needs to establish finely tuned calcium pathways in order to be able to differentiate multiple tasks, [1-3].

While it is known that neurons can actively change their shape upon neuronal activity, [4-7], we here present novel findings of activity-regulated nuclear morphology, [8,9]. With the help of an experimental and computational modeling approach, we show that hippocampal neurons can change the previously spherical shape of their nuclei to complex and infolded morphologies. This morphology regulation is demonstrated to be regulated by NMDA-receptor gated calcium, while synaptic and extra-synaptic NMDA-receptors elicit opposing effects on nuclear morphology, [8].

The structural alterations of the cell nucleus have significant effects on nuclear calcium dynamics. Compartmentalization of the nucleus, due to membrane infoldings, changes calcium frequencies, amplitudes and spatial distributions, [8,10]. Since these parameters have been shown to control downstream events towards gene transcription, [11,12], the results elucidate the cellular control of nuclear function with the help of morphology modulation. With respect to processes downstream of calcium, we show that histone H3 phosphorylation is closely linked to nuclear morphology. Investigating the nuclear morphologies of hippocampal neurons, two major classes were identified [9,10]. One class contains non-infolded nuclei that have the function of calcium signal integrators, while the other class contains highly infolded nuclei, which function as frequency detectors of nuclear calcium, [10].

Extending this interdisciplinary approach of investigating structure/function relationships in neurons, the effects of cellular morphology – as well as the morphology of the endoplasmic reticulum and other organelles – on neuronal calcium signals is currently being investigated. This endeavor makes use of highly detailed, three-dimensional models of neuronal calcium dynamics, including the three-dimensional morphology of the cell and its organelles.
